# Role of Hydrogen Sulfide in Retinal Diseases

**DOI:** 10.3389/fphar.2017.00588

**Published:** 2017-08-29

**Authors:** Jiantong Du, Hongfang Jin, Liu Yang

**Affiliations:** ^1^Department of Ophthalmology, Peking University First Hospital Beijing, China; ^2^Department of Pediatrics, Peking University First Hospital Beijing, China

**Keywords:** H_2_S, neuromodulator, physiology, pathology, retinal vascular diseases, retinal degenerative diseases

## Abstract

As the third gasotransmitter, hydrogen sulfide (H_2_S) plays a crucial role in the physiology and pathophysiology of many systems in the body, such as the nervous, cardiovascular, respiratory, and gastrointestinal systems. The mechanisms for its effects, including inhibiting ischemic injury, reducing oxidative stress damage, regulating apoptosis, and reducing the inflammation reaction in different systems, have not been fully understood. Recently, H_2_S and its endogenous synthesis pathway were found in the mammalian retina. This review describes the production and the metabolism of H_2_S and the evidence of a role of H_2_S in the retina physiology and in the different retinal diseases, including retinal degenerative diseases and vascular diseases. In the retina, H_2_S is generated in the presence of cystathionine-β-synthase, cystathionine-γ-lyase, and 3-mercaptopyruvate sulfurtransferase from L-cysteine. The role of endogenous H_2_S and its physiologic effect in the retina are still elusive. However, strong evidence shows that retina-derived H_2_S might play protective or deleterious role in the pathogenesis of retinal diseases. For example, by regulating Ca^2+^ influx, H_2_S can protect retinal neurons against light-induced degeneration. H_2_S preconditioning can mediate the anti-apoptotic effect of retinal ganglion cells in retinal ischemia/reperfusion injury. Treatment with H_2_S in rats relieves diabetic retinopathy by suppressing oxidative stress and reducing inflammation. Further studies would greatly improve our understanding of the pathophysiologic mechanisms responsible for retinal diseases and the potential for the H_2_S-related therapy of the retinal diseases as well.

## Introduction

Hydrogen sulfide (H_2_S), a well-known toxic gas characterized by its “rotten-egg” smell, has attracted substantial interest for its non-toxic effects in mammals. H_2_S was considered as an important gasotransmitter since Abe and Kimura (1996) discovered the enzymatic reaction process of H_2_S generation in the brain tissues along with its biological activity at a physiological concentration. Since then, the gasotransmitter H_2_S has been found to be involved physiologically and pathologically in the neuroregulation (Pérez-H et al., 2014), vasodilatation (Hosoki et al., 1997), endocrinologic regulation (Kaneko et al., 2006), and inflammation (Du et al., 2014), etc.

**FIGURE 1:**
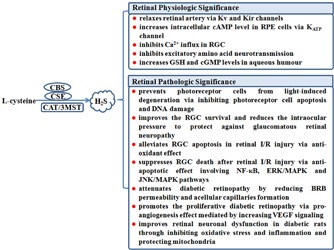
The role of endogenous H_2_S in the physiologic and pathophysiologic regulation in the retina. 3MST, 3-mercaptopyruvate sulfurtransferase; BRB, blood–retina barrier; cAMP, cyclic adenosine monophosphate; CAT, cysteine aminotransferase; CBS, cystathionine-β-synthase; cGMP, cyclic guanosine monophosphate; CSE, cystathionine-γ-lyase; ERK, extracellular signal regulated kinase; GSH, glutathione; H_2_S, hydrogen sulfide; I/R, ischemia/reperfusion; JNK, c-Jun N-terminal kinase; K_ATP_, ATP-sensitive potassium channel; K_ir_, inwardly rectifying potassium channel; K_v_, voltage-dependent potassium channel; MAPK, mitogen-activated protein kinase; NF-κB, nuclear factor-κB; RGC, retinal ganglion cell; RPE, retinal pigment epithelial; VEGF, vascular endothelial growth factor.

Recently, strong evidence has shown the presence of H_2_S and its endogenous synthesis pathway in the mammalian retina. This review presents the production of endogenous H_2_S and the evidence of its role in the retinal physiology and different retinal diseases including the retinal degenerative and vascular diseases, and the underlying mechanism (**Figure 1**).

## H_2_S Generation in the Retinal Tissues

So far, four enzymatic pathways that regulate endogenous H_2_S production have been revealed: cystathionine-β-synthase (CBS), cystathionine-γ-lyase (CSE) (Stipanuk and Beck, 1982; Abe and Kimura, 1996), cysteine aminotransferase/3-mercaptopyruvate sulfurtransferase (CAT/3MST) (Hughes et al., 2009) and D-amino acid oxidase (DAO)/3MST (Shibuya et al., 2013). However, only the first three endogenous H_2_S synthesis pathways have been reported to be involved in the retinal tissue, and there is no report of DAO/3MST pathways present in retinal tissue. The results of the distribution of the endogenous H_2_S synthesis and regulation in retinal tissue are controversial due to the different species and methods used in the study. Pong et al. (2007) found the expression and activity of CBS and CSE in the retinal tissue of salamander and those of CSE in the retinal tissue of mice. Mikami et al. (2011) detected the expression of endogenous H_2_S producing enzymes in each layer of the retina in mice by immunohistochemistry. The result showed that 3MST and CAT were expressed in the inner plexiform layer, outer plexiform layer, inner nuclear layer, outer nuclear layer, and outer segments of photoreceptors of the retina, with the absence of CBS and CSE, which suggests that H_2_S generation might be catalyzed almost by the CAT/3MST pathway in the mouse retina (Mikami et al., 2011). Gersztenkorn et al. (2016) further confirmed CBS, CSE, and 3MST protein expression in mouse retinal tissue by Western blot and immunohistochemistry. Kulkarni et al. (2011) used inhibitors of H_2_S-producing enzymes to study the endogenous H_2_S synthesis pathway in the bovine retina and the results showed that the use of CBS inhibitor or the use of CSE inhibitor, or the combined application of CBS and CSE inhibitors could not completely block the endogenous H_2_S generation in bovine retina, which indicated that perhaps other enzymes apart from CSE and CBE might contribute to H_2_S generation in bovine retina.

## Effect of H_2_S on Retinal Physiology

In the central nervous system (CNS), H_2_S has been reported to regulate synaptic activities as a neurotransmitter (Kimura et al., 2005). However, Qu et al. (2006) found that H_2_S acted as a mediator of ischemic injury on neurons and the inhibition of endogenous H_2_S production would be a potential neuroprotective strategy for stroke. Ion channels and transporters were found to be involved in the regulatory effects of H_2_S on CNS (Tang et al., 2010; Kimura, 2011). We have few studies of the physiologic effects of H_2_S in the retina, but the presence of H_2_S and its endogenous synthesis pathway in the retina as well as the fact that deficiency of CBS may lead to retinal degeneration and detachment (Kraus and Kozich, 2001) both indicate that H_2_S plays an important role in the eye as a gaseous neuromodulator.

### Effect of H_2_S on Ion Channels in the Retina

Potassium channels including ATP-sensitive potassium channel (K_ATP_) are important to the physiologic role of H_2_S (Zhao et al., 2001; Geng et al., 2004). Given the well-known regulatory effect of potassium channels in the retina (Cordeiro et al., 2011), it is reasonable to hypothesize that H_2_S modulates retinal function by acting on the potassium channel. Njie-Mbye et al. found that glibenclamide, a K_ATP_ channel inhibitor, inhibited the H_2_S donor NaHS-induced cyclic adenosine monophosphate (cAMP) formation in rat retinal pigment epithelial (RPE) cells, suggesting that K_ATP_-channel might be involved in the effect of H_2_S on the cAMP-related RPE cell proliferation, apoptosis, and phagocytosis (Njie-Mbye et al., 2012). NaHS was found to have a prominent relaxation effect on the retina arteries by acting on the voltage-dependent potassium channel (K_v_) and inwardly rectifying potassium channel (K_ir_) (Takır et al., 2015), indicating that H_2_S may play an important role in regulating the retina vascular system.

The calcium transport system exists in retinal Müller cells as well as RPE (Bringmann et al., 2000; Wimmers et al., 2006), and the change in these channels may contribute to the retinal degenerative diseases (Wimmers et al., 2006). Mikami et al. (2011) found that intracellular Ca^2+^ inhibited CAT/3MST-derived H_2_S production in retinal lysates and in turn H_2_S blocked high K^+^-induced Ca^2+^ influx via activating V-ATPase in the outer nuclear layer (ONL) and outer plexiform layer (OPL). The counteraction between the H_2_S and intracellular Ca^2+^ in the retina mediated the protective effect of H_2_S on the retinal photoreceptor apoptosis caused by excessive light (Mikami et al., 2011).

Besides K^+^ and Ca^2+^ channels, other ion channels such as sodium and chloride channels are believed to have an important effect on various physiologic processes in the retina (Zhang et al., 2011; Smith et al., 2017). However, whether H_2_S acts on the sodium and chloride channels has not been clear yet. Therefore, further studies need to be done to explore the possible relationship between retina-derived H_2_S and the ion channels.

### Regulation of Neurotransmission by H_2_S in the Retina

Glutamate is an important neurotransmitter and plays a key role in the fast excitatory synaptic transmission in the CNS. Therefore, glutamate has been reported to be implicated in the physiologic processes such as neuronal development and synaptic plasticity and pathophysiologic processes such as excitotoxicity (Seki et al., 2010). [^3^H]D-aspartate is a substitute for glutamate in neurotransmitter release assay. Opere et al. (2009) found that both NaHS and Na_2_S donors inhibited high K^+^-evoked [^3^H]D-aspartate release from isolated bovine and porcine retinae. Although the mechanisms were unclear, but it is supposed that there might be an involvement of glutamate aspartate transporter which lowers the extracellular glutamate level to protect the neurons against excitotoxic damage. One study suggested that a derivative of latanoprost acid (ACS67), which can release H_2_S, had a remarkable effect on increasing glutathione (GSH) and cyclic guanosine monophosphate (cGMP) levels in the aqueous humor (Perrino et al., 2009). In the retina, the glutamate aspartate transporter is located in Müller cells and maintains the level of GSH with the toxic effects of glutamate (Martin et al., 2012). Therefore, this transporter might be a target of H_2_S to regulate neurotransmission in the retina. Future studies of the exact mechanisms are needed.

## Effect of H_2_S on Retinal Pathology

Retinal diseases including retinal degenerative diseases and vascular diseases have become the leading causes of blindness reported by the World Health Organization (Pascolini and Mariotti, 2012). Their irreversible damage to vision and refractory characteristics make them a hot topic for ophthalmologists all over the world. Here, we discuss the effects of H_2_S on retinal degenerative diseases and vascular diseases.

### H_2_S and Retinal Degenerative Diseases

Retinal degenerative diseases, commonly including retinitis pigmentosa (RP), age-related macular degeneration (AMD), and glaucomatous retinal neuropathy, share the main pathological basis of abnormal structure and function of retinal neurons at all levels, which results in an irreversible damage to visual acuity (Cottet and Schorderet, 2009).

Progressive degeneration of the photoreceptor cells in the retina contributes to the severe visual injury of RP and AMD (Busskamp et al., 2010). The photoreceptor cell apoptosis is a key pathologic basis of human retinal degeneration and light-induced retinal degeneration models (Wenzel et al., 2005; Rajala and Rajala, 2013). In one study, NaHS administration suppressed the light-induced photoreceptor degeneration in association with decreasing photoreceptor cell apoptosis and DNA damage in the retinal ONL in a mice retinal degeneration model caused by excessive light exposure. Simultaneously, NaHS prevented high K^+^-evoked Ca^2+^ influx in mice retinal ONL and OPL (Mikami et al., 2011), suggesting that restoring Ca^2+^ homeostasis might be involved in the protective effect of H_2_S on the photoreceptor cell apoptosis. The above findings indicate that H_2_S might act as a neuroprotector to prevent from retinal degeneration. On the other hand, abnormal function of RPE cells is an important pathological process of RP and AMD (Da et al., 2007; Liu et al., 2015). H_2_S donor NaHS and endogenous H_2_S were found to dose-dependently increase cAMP concentrations in rat RPE cells (Njie-Mbye et al., 2010), while the increase in intracellular cAMP level resulted in the downregulation of phagocytic activity of RPE cells, which would lead to the progression of RP and AMD (Hall et al., 1993; Kuriyama et al., 1995). The above facts suggest that H_2_S might aggravate the process of RP and AMD. Collectively, whether H_2_S has a protective or deleterious effect on RP and AMD needs further research.

Although glaucoma is not usually described as a retinal disease, glaucomatous retinal neuropathy is the leading cause of vision impairment in glaucoma (Low et al., 2015). Recently, H_2_S levels and expression of its endogenous enzymes CBS, CSE, and 3MST in retinal tissues were significantly decreased along with the loss of retinal ganglion cells (RGCs) in a chronic ocular-hypertension rat model. While the treatment with NaHS could improve the survival of RGCs without impacting on the intraocular pressure (Huang et al., 2017). However, Perrino et al. (2009) found that H_2_S-releasing agent ACS67 reduced the intraocular pressure in carbomer-induced glaucoma in rabbits and increased the GSH and cGMP content in aqueous humor. The above interesting results give a better understanding of the pathogenesis of glaucomatous retinal neuropathy and provide a potential therapeutic target for glaucoma.

### H_2_S and Retinal Vascular Diseases

Retinal vascular diseases are defined as diseases first caused by a retinal vascular abnormality, which leads to injured retinal neurons or vision. The diseases mainly include retinal artery occlusion (RAO), retinal vein occlusion (RVO), and diabetic retinopathy (DR).

In retinal vascular occlusion, including RAO and RVO, ischemia/reperfusion (I/R) injury plays a significant role in the pathologic process (Archer, 1976). It causes RGCs death by inducing apoptosis and necrosis (Hayreh et al., 2004). Intravitreal injection of the H_2_S donor ACS67 could prevent the retinal injury caused by elevated intraocular pressure-induced retina I/R in an *in vivo* experiment. Furthermore, *in vitro* study showed that ACS67 suppressed H_2_O_2_-induced RGC-5 cell apoptosis and improved RGC-5 cell viability via increasing the GSH level and decreasing reactive oxygen species level (Osborne et al., 2010). Biermann et al. (2011) found that inhaled H_2_S preconditioning attenuated RGC death after retina I/R injury. NF-κB, ERK/MAPK, and JNK/MAPK pathways were involved in the anti-apoptotic mechanisms for H_2_S preconditioning (Biermann et al., 2011). Additionally, H_2_S could relax retinal arteries by targeting on the potassium channel (Takır et al., 2015), which might be involved in the protective effect of H_2_S on the retinal vascular diseases.

DR, due to diabetes, is a globally increasingly important retinal disease (Whiting et al., 2011). High glucose-induced retinal microvasculopathy is one of the important causes of reduced visual acuity and acquired blindness. The injured pericytes and capillary blockage due to a high glucose environment, which further leads to generation of vascular endothelial growth factor (VEGF), are the most critically early pathological changes during DR (Lu and Adamis, 2002). The process further leads to the breakdown of the blood–retinal barrier, leakage of retinal capillaries, macular edema, and neovascularization. In streptozotocin-induced diabetic rats, retina H_2_S level and CSE and 3MST mRNA were decreased. The treatment of NaHS could reduce blood–retinal barrier permeability and acellular capillaries formation in the retina along with the reduced vitreous content of VEGF and its signaling pathway (Si et al., 2013), suggesting that retina-derived H_2_S might protect against high glucose-induced retinal microvasculopathy. However, in another study, H_2_S was found to play different roles in the development of retinal neovascularization in diabetic patients with proliferative DR (PDR). Ran et al. (2014) revealed that H_2_S level was increased in both the plasma and the vitreous body of diabetic patients with PDR when compared with that in healthy people and diabetic patients without PDR. In accordance with the increase in H_2_S level, VEGF levels in the vitreous body from diabetic patients with PDR were also significantly elevated. The above clinical data suggest that H_2_S might act as a pro-angiogenesis factor to promote the progression of PDR (Ran et al., 2014), which is similar to the role of H_2_S in the development of angiogenesis in cardiovascular diseases (Cai et al., 2007; Papapetropoulos et al., 2009; Wang et al., 2010). Recently, retina neurodegeneration was found to occur ahead of vascular injury during DR (Aizu et al., 2003; Ozkaya et al., 2017). Si et al. (2013) found that NaHS improved retinal neuronal dysfunction in streptozotocin-treated rats representing the facts that NaHS enhanced b-wave amplitudes and oscillatory potentials. The underlying mechanisms responsible for the neuroprotective effect of H_2_S in retina included suppressing oxidative stress, protecting mitochondrial function, and inhibiting inflammation. Taken together, H_2_S plays a complex role in the pathogenesis of DR. Further studies are needed to discover the effect and mechanisms for H_2_S in DR, which might hopefully deepen the understanding of the DR pathophysiology and help to find new treatments for this disease.

## Conclusion

Since H_2_S and its endogenous synthesis pathway were found in mammalian retina, studies about the effect of H_2_S on retina physiology and pathology have become a hot topic for ophthalmologists (**Figure 1**). In the past years, H_2_S was proved as a neuromodulator in the eye with important effects in some retinal diseases. However, the role of H_2_S in lots of retinal diseases still needs to be revealed and in-depth studies of the underlying mechanisms are bound to be necessary. As we know, unlike CNS or cardiovascular system, the unique characteristics of retina is the direct connection to the vitreous body, which is a perfect match to gaseous treatment that has been widely used in the research of many retina diseases like retinal detachment and macular membrane. Therefore, the H_2_S-related therapy has a bright future but still requires further studies.

## Author Contributions

The manuscript was written through contributions of all authors. All authors have given approval to the final version of the manuscript. JD and HJ researched and identified appropriate articles. JD participated in writing the manuscript. HJ and LY revised the manuscript.

## Conflict of Interest Statement

The authors declare that the research was conducted in the absence of any commercial or financial relationships that could be construed as a potential conflict of interest.
